# Endoscopic and Histopathological Characteristics of Newly Diagnosed Crohn's Disease in Vietnam: Insights From a Tertiary IBD Center

**DOI:** 10.1002/jgh3.70488

**Published:** 2026-10-23

**Authors:** Trung Hoang Dinh, Luan Minh Dang, Chuong Dinh Nguyen, Diem Thi‐Ngoc Vo, Thong Duy Vo, Hoang Huu Bui, Duc Trong Quach

**Affiliations:** ^1^ Department of Internal Medicine Nam Sai Gon International General Hospital Ho Chi Minh City Vietnam; ^2^ Department of Gastroenterology University Medical Center Ho Chi Minh City Ho Chi Minh City Vietnam; ^3^ Department of Internal Medicine and Gastro‐Hepato Integrated Research Team (GHIRT‐002.TCM2025) School of Medicine, University of Medicine and Pharmacy at Ho Chi Minh City Ho Chi Minh City Vietnam; ^4^ Faculty of Medicine Chulalongkorn University Bangkok Thailand; ^5^ Department of Histology‐Embryology and Pathology School of Medicine, University of Medicine and Pharmacy at Ho Chi Minh City Ho Chi Minh City Vietnam

**Keywords:** Crohn's disease, endoscopy, histopathology, inflammatory bowel disease, Southeast Asia, Vietnam

## Abstract

**Background and Aims:**

Crohn's disease (CD) is rapidly emerging in Asia, yet endoscopic and histopathological characteristics at diagnosis remain poorly described in Southeast Asia. We aimed to describe baseline endoscopic and histologic features in Vietnamese adults with newly diagnosed CD.

**Methods:**

We conducted a descriptive cross‐sectional study of consecutive adults newly diagnosed with CD from June 2019 to June 2024 at a tertiary inflammatory bowel disease (IBD) center in Vietnam. Ileocolonoscopy with segmental biopsies was performed in all patients. Endoscopic findings, histopathology, and Montreal classification were systematically recorded.

**Results:**

Eighty patients (median age 34.5 years, 62.5% male) were included. The median time from symptom onset to diagnosis was 12 months (IQR 2–24); 78.8% had ≥ 1 prior inconclusive colonoscopy, and 25% had undergone intestinal resection before confirmed diagnosis. Ileocolonic disease (L3) predominated (71.3%). At diagnosis, 28.8% of patients had stricturing and 28.8% had penetrating disease behavior. Endoscopically, terminal ileal involvement was observed in 77.6%, cecal involvement in 56.6%, and skip lesions in 72.4%. Aphthous ulcers were seen in 13.2%, longitudinal ulcers in 14.5%, and cobblestoning in 6.6%. Luminal narrowing and fistulous openings were seen in 32.9% and 5.3%, respectively. The overall endoscopic impression was consistent with CD in 46.1%, non‐specific in 35.5%, suggestive of colorectal cancer (CRC) in 13.2%, and suggestive of intestinal tuberculosis in 3.9%. Histologically, chronic inflammation was universal and non‐caseating epithelioid granulomas were identified in 31.6%. The pathologist's conclusion was consistent with CD in 67.1% and non‐specific in 30.4%.

**Conclusions:**

In this tertiary‐center cohort of Vietnamese adults with newly diagnosed CD, the median time from symptom onset to diagnosis was 12 months. At diagnosis, 57.5% had stricturing or penetrating behavior, and 25.0% had already undergone intestinal resection. Longitudinal ulcers, cobblestoning, and epithelioid granulomas were each found in a minority of patients, and histopathology was nonspecific in 30.4%. These descriptive data provide a baseline for future comparative studies and for evaluating standardized diagnostic criteria in Vietnam.

**Abbreviations**
CD: Crohn's disease
CRC: colorectal cancer
ECCO: European Crohn's and Colitis Organization
EGD: esophagogastroduodenoscopy
IBD: inflammatory bowel disease
IQR: interquartile range
TB: tuberculosis

## 1 Introduction

Crohn's disease (CD) is a chronic, transmural inflammatory disorder that can affect any segment of the gastrointestinal tract from mouth to anus. It is characterized by a relapsing–remitting course and progressive bowel damage leading to strictures, fistulae, and disability [1]. Once regarded as a disease predominantly of Western countries, CD has shown a dramatic rise in incidence and prevalence across Asia over the past three decades [2, 3, 4]. For instance, a population‐based study from South Korea has documented a 40‐fold increase in CD incidence between the 1980s and 2010s, with current prevalence reaching approximately 31.6 per 100 000 inhabitants [5]. By contrast, Southeast Asia remains in the early phases of the four epidemiological stages of inflammatory bowel disease (IBD), although recent data confirm a clear and accelerating upward trend in CD incidence in Thailand, Malaysia, and Singapore [6]. The rising prevalence likely reflects a combination of true increases in incidence, greater physician awareness, and improved diagnostic capabilities [4, 6].

Vietnam is currently classified as a country in the emergence stage, with rising incidence of reported cases [3]. However, no comprehensive data on endoscopic and histopathological phenotypes at diagnosis have been published from Vietnam. Our recent multinational, multicenter study comparing newly diagnosed Vietnamese and Korean patients demonstrated significantly higher disease severity and a greater proportion of complicated (stricturing and penetrating) behavior in CD among Vietnamese patients at diagnosis, findings which are largely attributable to delayed recognition in a low‐awareness and high‐tuberculosis (TB)‐burden setting [7].

Accurate diagnosis of CD relies heavily on characteristic endoscopic and histopathological findings [8]. Classical endoscopic features include discontinuous (skip) lesions, longitudinal ulcers, cobblestoning, presence of strictures, and perianal involvement, whereas hallmark histological findings comprise focal or patchy chronic inflammation, crypt architectural distortion, and noncaseating epithelioid granulomas, which are detected in only 10%–40% of CD patients [8, 9, 10].

Despite the increasing burden of CD in Vietnam, no large single‐center series has systematically described baseline endoscopic and histopathological characteristics in southern Vietnamese patients, where rapid urbanization and dietary westernization may drive unique phenotypic shifts. Establishing such baseline data is crucial, not only for improving diagnostic accuracy and differentiation from intestinal TB in a high‐burden setting, but also for defining local benchmarks essential to implement “treat‐to‐target” strategies and facilitate precise phenotypic comparisons within Asia.

We therefore conducted a combined retrospective and prospective descriptive study of 80 consecutive adults with newly diagnosed CD at the IBD Unit, University Medical Center Ho Chi Minh City, one of the largest tertiary referral centers for IBD in Vietnam, to detail the endoscopic and histopathological features at diagnosis and to describe these findings in the context of published Asian and Western reports.

## 2 Methods

### 2.1 Study Design and Context

This was a descriptive cross‐sectional study evaluating the baseline characteristics of consecutive adult patients newly diagnosed with CD at the IBD Unit, Department of Gastroenterology, University Medical Center Ho Chi Minh City. The study cohort was assembled through bidirectional data collection encompassing a 5‐year period: a retrospective review of electronic medical records (June 2019–November 2023) and a prospective enrollment phase using standardized case report forms (November 2023–June 2024). Written informed consent was obtained from all prospectively enrolled patients; consent was waived for retrospective cases per ethics committee approval. The study was approved by the Ethics Committee of University of Medicine and Pharmacy at Ho Chi Minh City (approval number: 711/HDDD‐DHYD, date: 15 May 2023).

### 2.2 Study Participants and Data Collection

All consecutive adult patients (aged ≥ 18 years) with newly diagnosed CD during the study period were included. CD was diagnosed according to current European Crohn's and Colitis Organization (ECCO) guidelines using a combination of clinical, endoscopic, radiological, and histological criteria [8]. All endoscopic procedures were performed by gastroenterologists experienced in IBD. During ileocolonoscopy, our institutional protocol aimed to take at least two biopsy specimens from a minimum of five distinct sites, encompassing both endoscopically abnormal lesions and normal‐appearing mucosa, in accordance with current ECCO guidelines [8]. In cases where severe luminal strictures precluded scope passage, biopsies were obtained from as many accessible segments as possible. Histopathological examinations were reviewed by experienced gastrointestinal pathologists specializing in IBD. For prospectively enrolled patients, findings were recorded using a standardized case report form; for retrospective cases, data were extracted from medical records using a predefined data collection template. To minimize information bias in the retrospective cohort, data extraction was not strictly limited to written medical reports. All available stored endoscopic images were independently re‐reviewed by experienced IBD gastroenterologists (L. M. D., C. D. N., and T. D. V.). Any discrepancies in endoscopic interpretation were resolved through discussion and consensus with a senior endoscopist (D. T. Q.). Similarly, archived histopathological slides from retrospective cases were re‐evaluated by an expert gastrointestinal pathologist (D. T. N. V.) to ensure a standardized assessment of microscopic features according to current diagnostic criteria. At our institution, the standard diagnostic work‐up for all suspected CD patients routinely incorporates cross‐sectional imaging, specifically computed tomography enterography (CTE) or magnetic resonance enterography (MRE) to comprehensively evaluate small bowel involvement. However, as the primary objective of this current study is specifically to characterize the macroscopic (endoscopic) and microscopic (histopathological) phenotypes, detailed radiological parameters and their specific correlations fall outside the scope of this analysis. Given the endemic setting, intestinal tuberculosis was excluded using a combination of clinical, radiologic, and microbiological evaluation, including chest radiography, Interferon‐Gamma Release Assay (IGRA), and tissue testing with mycobacterial culture and polymerase chain reaction (PCR) when clinically indicated. In cases with ambiguous histology where an empirical anti‐tubercular therapy (ATT) trial was administered, a lack of clinical and endoscopic response did not automatically equate to a CD diagnosis. Instead, non‐responders underwent a rigorous clinical re‐evaluation to exclude confounders such as poor medication compliance, inadequate dosing, or multidrug‐resistant TB (MDR‐TB). A definitive CD diagnosis was subsequently established only after repeating the comprehensive diagnostic work‐up, including endoscopy and histology, to confirm features consistent with CD. Only patients with definite CD were enrolled. Exclusion criteria were: (1) patients already diagnosed and treated at other institutions before referral; (2) IBD‐unclassified cases after complete work‐up; (3) isolated perianal disease without luminal involvement; and (4) incomplete diagnostic evaluation precluding definitive diagnosis.

Medical records were systematically reviewed. The following baseline data at the time of diagnosis were extracted:

- Demographic and clinical characteristics (age at diagnosis, sex, smoking status, family history of IBD, duration from symptom onset to diagnosis, Montreal classification [A1–A3, L1–L4, B1–B3 ± *p*]).
- Endoscopic findings: procedure type (ileocolonoscopy ± esophagogastroduodenoscopy [EGD]), extent of examination (terminal ileum intubated or not), presence and location of mucosal lesions (as listed in Table 2), and the overall endoscopic impression (see Definitions).
- Histopathological findings from all available biopsies (ileum, colon, upper gastrointestinal (GI) if performed): presence of chronic inflammation, focal/discontinuous inflammation, crypt architectural distortion, granulomas (epithelioid ± giant cells, non‐caseating), pyloric gland metaplasia, neuronal hyperplasia, and final pathologist's conclusion (consistent with CD, nonspecific chronic inflammation, or suggestive of infection). This conclusion was a global expert assessment. It was reported as “consistent with CD” when the combination of features favored CD over other causes of chronic inflammation. Such features included focal/discontinuous inflammation, focal crypt distortion, and non‐caseating epithelioid granulomas. Chronic inflammation without such features was reported as non‐specific. No validated scoring system for mucosal biopsies was applied. All biopsies were reviewed by experienced gastrointestinal pathologists at our institution using hematoxylin–eosin staining; special stains for acid‐fast bacilli and fungi were performed when clinically indicated.

### 2.3 Definitions

Disease location and behavior were classified according to the Montreal classification. Perianal disease modifier (+p) was recorded if fistulae, abscesses, or anal ulcers were present at diagnosis or within 3 months. Skip lesions were defined as segments of inflamed mucosa separated by normal‐appearing intervening mucosa. The overall endoscopic impression was assigned to one of five mutually exclusive categories. “Consistent with CD” required predominant macroscopic features characteristic of CD, based on ECCO criteria (e.g., longitudinal ulcers, cobblestoning, or aphthous or discrete ulcers with a skip distribution) [8]. “Non‐specific inflammatory changes” denoted erythema, erosions, and/or ulcers without these features and without features of another diagnosis. “Suggestive of CRC” denoted an ulcerated mass‐like lesion or an irregular, ulcerated circumferential narrowing suspicious for malignancy. “Suggestive of intestinal TB” denoted predominant features favoring TB, such as transverse or circumferential ulcers or a patulous ileocecal valve. Luminal narrowing and fistulous openings were recorded separately as lesion‐level findings (Table 2) and did not by themselves determine the impression category. Disease behavior (B1–B3) was assigned by the Montreal classification, using all endoscopic, radiological, and surgical information. Granuloma was defined as a well‐formed collection of epithelioid histiocytes with or without multinucleated giant cells and without caseation. Diagnostic delay was defined as the time interval (in months) from the patient's recall of the onset of initial CD‐related gastrointestinal symptoms (e.g., chronic abdominal pain, diarrhea, or weight loss) to the date of definitive CD diagnosis.

### 2.4 Statistical Analysis

Statistical analysis was performed using IBM SPSS version 25.0 (IBM Corp., Armonk, NY, USA). Continuous variables with non‐normal distribution were expressed as median and interquartile range (IQR) and compared using the Mann–Whitney U test. Categorical variables were presented as absolute numbers and percentages and compared using χ^2^ test or Fisher's exact test as appropriate. All tests were two‐sided, and a *p* value < 0.05 was considered statistically significant. No adjustments were made for multiple comparisons given the descriptive and exploratory nature of this study.

## 3 Results

From June 2019 to June 2024, a total of 126 consecutive adult patients were newly diagnosed with inflammatory bowel disease at the IBD Unit, University Medical Center Ho Chi Minh City. Of these, 80 patients (63.5%) fulfilled the criteria for definite CD and were included in the present analysis; no patients were classified as IBD‐unclassified (Figure 1).

**FIGURE 1:**
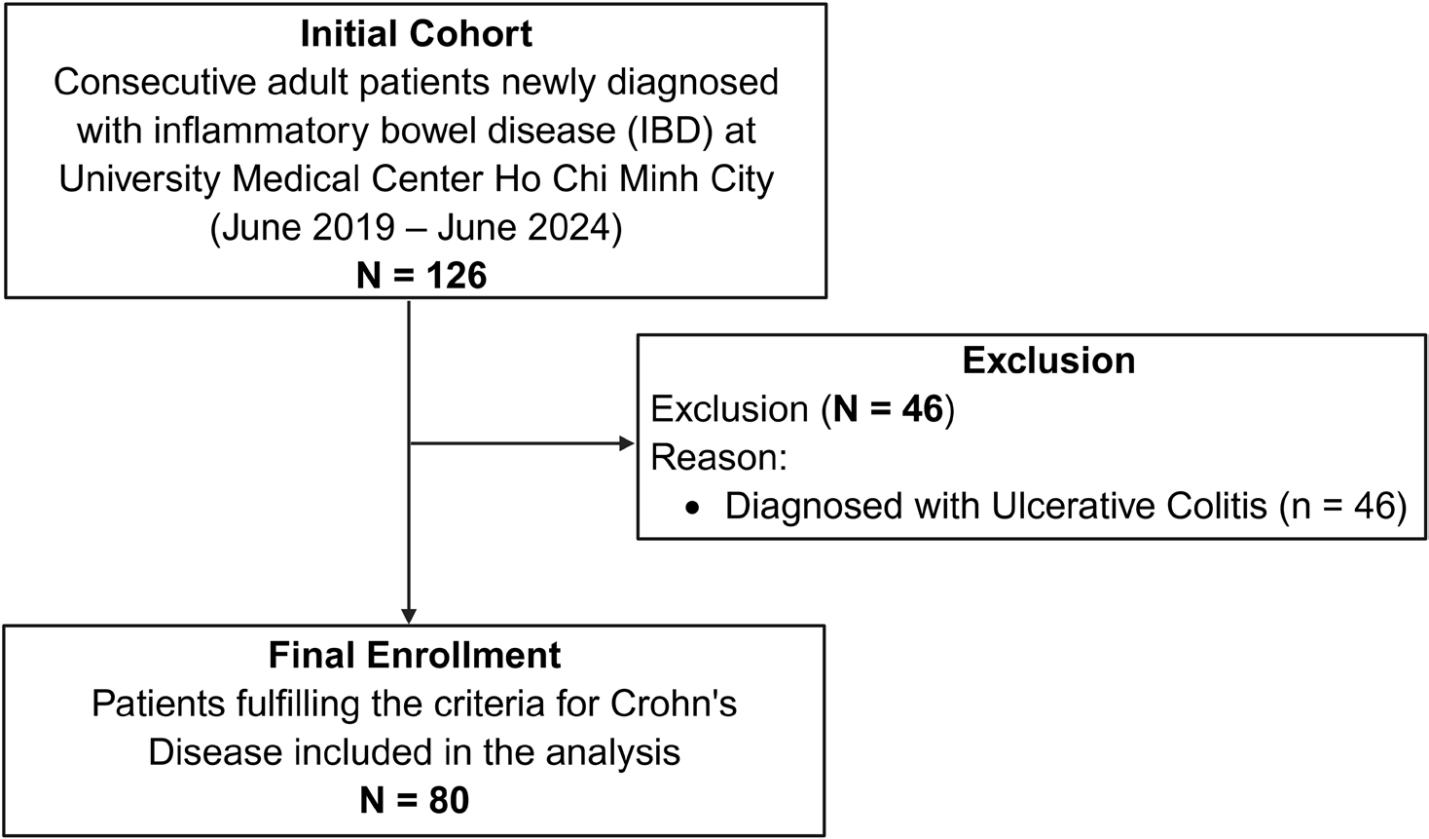
Study flowchart.

### 3.1 Baseline Characteristics

The baseline characteristics are presented in Table 1. The median age at diagnosis was 34.5 years (IQR 25–48) with male predominance (62.5%). The clinical presentation was characterized primarily by abdominal pain (87.5%) and diarrhea (85%). Systemic manifestations such as weight loss or fever were present in 36.3% and 5% of cases, respectively. Hematochezia was noted in 15.0% of patients. The median duration from symptom onset to diagnosis was 12 months (IQR 2–24), with 78.8% having undergone at least one prior colonoscopy (median 2 procedures). Notably, 20 patients (25.0%) had undergone intestinal resection prior to definitive diagnosis.

**TABLE 1:** Baseline demographic and clinical characteristics of 80 patients with newly diagnosed Crohn's disease.

Characteristics	Values
Age at diagnosis, median (IQR), years	34.5 (25–48)
Male sex, *n* (%)	50 (62.5)
Smoking status, *n* (%)	
Never	60 (75)
Ex‐smoker	14 (17.5)
Current smoker	6 (7.5)
Family history of IBD, *n* (%)	0 (0)
Duration from symptom onset to diagnosis, median (IQR), months	12 (2–24)
Symptoms, *n* (%)	
Abdominal pain	70 (87.5)
Diarrhea	68 (85)
Hematochezia	12 (15)
Weight loss	29 (36.3)
Fever	4 (5)
Prior colonoscopy, *n* (%)	63 (78.8)
Number of previous colonoscopies, median (IQR)	2 (1–2)
Empirical anti‐TB therapy, *n* (%)	9 (11.3)
Intestinal resection, *n* (%)	20 (25)
Montreal A: Age at diagnosis	
A1 (≤ 16 years), *n* (%)	0
A2 (17–40 years), *n* (%)	52 (65.0)
A3 (> 40 years), *n* (%)	28 (35)
Montreal L: Location^a^	
L1 (ileal), *n* (%)	12 (15)
L2 (colonic), *n* (%)	11 (13.8)
L3 (ileocolonic), *n* (%)	57 (71.3)
Upper GI involvement (L4), *n* (%)	4 (5)
Montreal B: Behavior^a^	
B1 (non‐stricturing non‐penetrating), *n* (%)	34 (42.5)
B2 (stricturing), *n* (%)	23 (28.8)
B3 (penetrating), *n* (%)	23 (28.8)
Perianal disease modifier (+p), *n* (%)	11 (13.8)

*Note:* Data are presented as number (percentage) or median (interquartile range [IQR]). IBD: inflammatory bowel disease; TB: tuberculosis; GI: gastrointestinal.

^a^ Not mutually exclusive.

Regarding Montreal classification, most patients were diagnosed between ages 17 and 40 years (A2, 65.0%). Ileocolonic involvement (L3) was most common (71.3%), with upper gastrointestinal involvement (L4) in 5.0%. Disease behavior showed 42.5% with inflammatory phenotype (B1), while stricturing (B2), and penetrating (B3) each accounted for 28.8%. Perianal disease was present in 13.8% at diagnosis.

### 3.2 Endoscopic Findings

#### 3.2.1 Ileocolonoscopy

Of the 80 patients with CD, 76 (95.0%) underwent ileocolonoscopy at diagnosis. Four patients did not undergo colonoscopy: three required emergency surgery prior to endoscopy (two for fistulizing disease and one for suspected malignant colonic obstruction on computed tomography [CT]), and one had a severe rectal stricture precluding scope passage.

Table 2 summarizes the detailed endoscopic findings. The predominant mucosal lesions were ulceration (63.2%) and erythema (61.8%). Aphthous ulcers, longitudinal ulcers, and cobblestoning were observed in 13.2%, 14.5%, and 6.6% of patients, respectively. Pseudopolyps were noted in 9.2% of cases.

**TABLE 2:** Endoscopic characteristics, anatomic distribution, and diagnostic impressions in 76 patients with Crohn's disease at diagnosis.

Endoscopic characteristics	*n* (%)
Endoscopic features^a^	
Mucosal erythema	47 (61.8)
Erosion	31 (40.8)
Ulceration (any type)	48 (63.2)
Aphthous ulcers	10 (13.2)
Longitudinal ulcers	11 (14.5)
Ulcerated mass	7 (9.2)
Cobblestoning	5 (6.6)
Pseudopolyps	7 (9.2)
Luminal narrowing	25 (32.9)
Fistulous opening	4 (5.3)
Distribution pattern	
Single‐segment involvement	21 (27.6)
Multisegment involvement with skip lesions	55 (72.4)
Anatomic location^a^	
Anal canal	12 (15.8)
Rectum	24 (31.6)
Sigmoid colon	32 (42.1)
Descending colon	20 (26.3)
Transverse colon	21 (27.6)
Ascending colon	21 (27.6)
Cecum	43 (56.6)
Terminal ileum	59 (77.6)
Surgical anastomosis^a^	6 (7.9)
Endoscopic impression	
Consistent with Crohn's disease	35 (46.1)
Suggestive of colorectal cancer	10 (13.2)
Suggestive of intestinal tuberculosis	3 (3.9)
Non‐specific inflammatory changes	27 (35.5)
Normal examination^b^	1 (1.3)

*Note:* Endoscopic impression categories are mutually exclusive and total 76 patients (100%); definitions are given in the Methods of the manuscript. Luminal narrowing and fistulous openings were recorded as lesion‐level findings, independent of the overall impression. Data are presented as number (percentage). CD: Crohn's disease; TB: Tuberculosis.

^a^ Not mutually exclusive.

^b^ Patient had isolated small bowel Crohn's disease confirmed by imaging.

Luminal narrowing was observed in 32.9% of patients, and fistulous openings were identified in 5.3%. An ulcerated mass was seen in 7 patients (9.2%). Representative endoscopic findings are shown in Figure 2. A surgical anastomosis was present in 6 patients (7.9%) with prior resection. Using the Montreal classification, which also incorporated imaging and surgical findings, 23 patients (28.8%) had stricturing (B2) and 23 (28.8%) had penetrating (B3) behavior (Table 1). Thus, penetrating complications were identified mainly by imaging or at surgery rather than at endoscopy.

**FIGURE 2:**
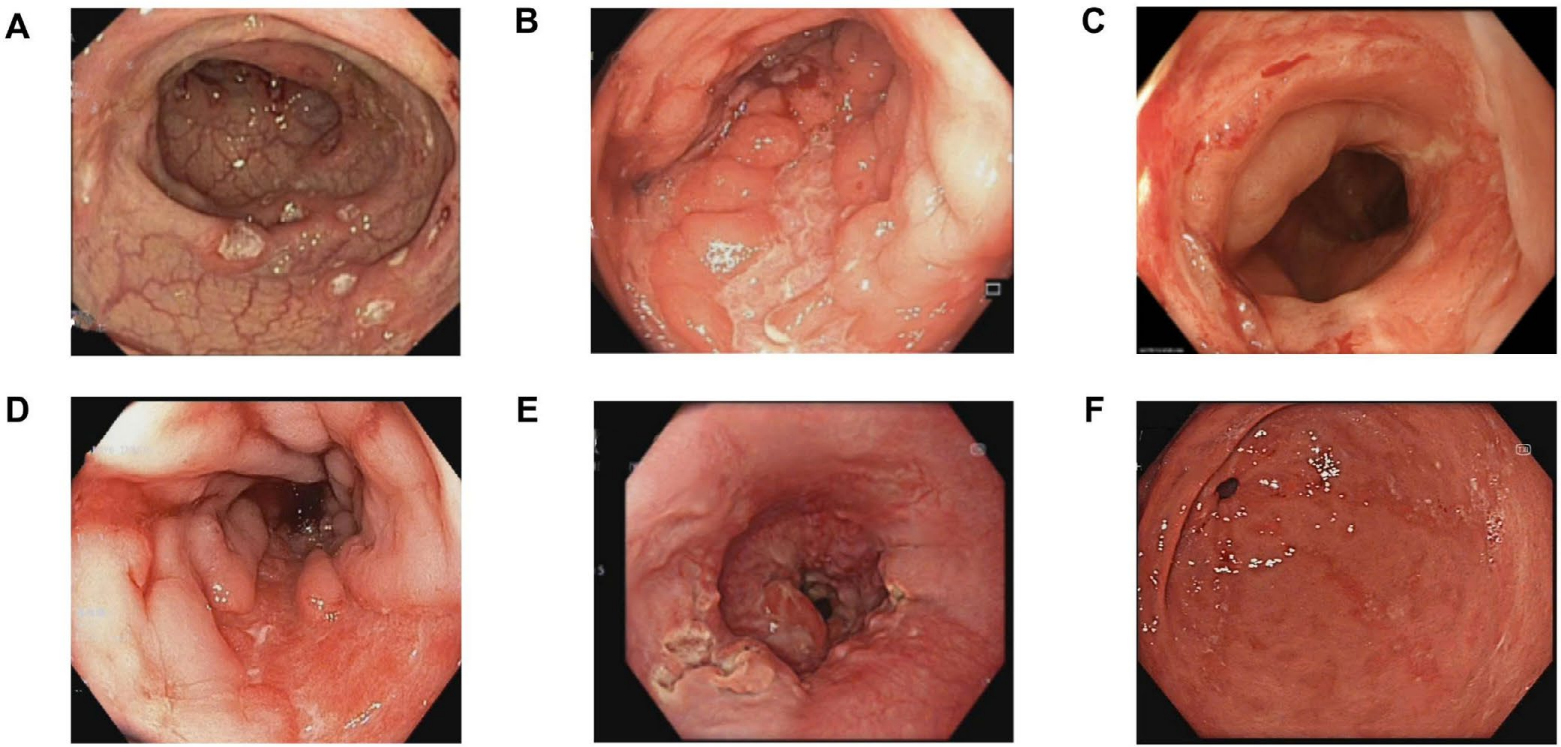
*Representative endoscopic images of newly diagnosed Crohn's disease in the study cohort*. (A) Aphthous ulcers in the colon: Small, discrete superficial ulcers surrounded by erythematous halos, arranged in a longitudinal pattern. (B) Typical “cobblestoning” appearance in the colon: Residual islands of edematous mucosa separated by intersecting deep longitudinal ulcerations. (C) Anal ulcer: A discrete ulceration in the perianal region. (D) Longitudinal ulceration in the colon associated with luminal stenosis: Deep, linear ulcers leading to narrowing of the intestinal lumen. (E) Deep ulceration in the esophagus: An ulcer confirmed by biopsy as consistent with Crohn's disease involvement. (F) Erosions in the gastric antrum: Superficial mucosal erosions with surrounding erythema, biopsy‐proven to be compatible with Crohn's disease.

Regarding the distribution of lesions: the ileocecal region was the most frequently affected site, terminal ileal involvement was observed in 77.6% of patients, and cecal involvement in 56.6%. Notably, 15.8% of patients had anal canal lesions. Regarding the disease pattern, 72.4% of patients exhibited multisegmental involvement characterized by skip lesions, consistent with the hallmark discontinuous nature of CD, whereas 27.6% presented with single‐segment disease.

Endoscopic impression: based on macroscopic appearance alone, the overall impression was consistent with CD in 35 patients (46.1%) and showed nonspecific inflammatory changes in 27 (35.5%). It was suggestive of CRC in 10 (13.2%) and of intestinal TB in 3 (3.9%); definitions are given in the Methods. In all patients with lesions suggestive of CRC, histopathology excluded malignancy and confirmed CD‐related lesions. One patient (1.3%) had a normal colonoscopy but demonstrated small‐bowel involvement on cross‐sectional imaging.

#### 3.2.2 Upper Gastrointestinal Endoscopy

Among the 80 patients, 38 (47.5%) underwent EGD due to upper GI symptoms. Lesions suggestive of CD were identified in 4 patients (5.0%), comprising duodenal ulcers (*n* = 2), gastric ulcer (*n* = 1), and combined gastric and esophageal ulceration (*n* = 1).

### 3.3 Histopathological Findings

Histopathological examination was performed in 79 of 80 patients (98.8%); one patient with severe rectal stricture could not undergo biopsy and did not require surgery. Of the 79 patients, 91.1% had biopsies obtained endoscopically, and 8.9% from surgical specimens.

Regarding microscopic features (Table 3), chronic inflammation was universally present (100%). The most common finding was basal plasmacytosis (73.4%), followed by crypt architectural distortion (38.0%), focal/discontinuous inflammation (26.6%), and transmural inflammation (35.4%). Non‐caseating epithelioid granulomas, a hallmark histological feature, were identified in 31.6% of patients overall. Transmural inflammation was significantly more frequent in surgical specimens than in endoscopic biopsies (85.7% vs. 30.6%, *p* = 0.007), as expected given the depth of sampling.

**TABLE 3:** Comparison of histopathological features and diagnostic yield between endoscopic biopsies and surgical resection specimens.

Histological features	Overall (*n* = 79)	Endoscopic biopsies (*n* = 72)	Surgical resection specimens (*n* = 7)	*p*
Chronic inflammation	79 (100%)	72 (100%)	7 (100%)	—
Basal plasmacytosis	58 (73.4%)	53 (73.6%)	5 (71.4%)	1.000
Crypt distortion	30 (38.0%)	26 (36.1%)	4 (57.1%)	0.417
Focal/discontinuous inflammation	21 (26.6%)	18 (25.0%)	3 (42.9%)	0.375
Epithelioid non‐caseating granuloma	25 (31.6%)	22 (30.6%)	3 (42.9%)	0.673
Transmural inflammation	28 (35.4%)	22 (30.6%)	6 (85.7%)	**0.007**
Pathologist's conclusion				
Consistent with Crohn's disease	53 (67.1%)	46 (63.9%)	7 (100%)	0.089
Non‐specific chronic inflammation	24 (30.4%)	24 (33.3%)	0 (0%)	—
Suggestive of Infection	2 (2.5%)	2 (2.8%)	0 (0%)	—

*Note:* Data are presented as number (percentage). CD: Crohn's disease. Transmural inflammation: Inflammation extending through the muscularis propria (assessable primarily in resection specimens). *p* values were calculated using Fisher's exact test comparing endoscopic biopsies versus surgical resection specimens (significance threshold *p* < 0.05). The bold formatting is intentional, as the *p*‐value (*p* = 0.007) indicates statistical significance.

Based on histopathology, the pathologist's conclusion in the 79 patients with histological data was consistent with CD in 53 (67.1%) and showed nonspecific chronic inflammation in 24 (30.4%). In 2 patients (2.5%), the findings were suggestive of infection pending its exclusion. No cases were conclusively diagnosed as TB or malignancy on histology. All included patients had a final diagnosis of CD. These proportions therefore describe histological findings in confirmed CD, not the diagnostic accuracy of histopathology.

## 4 Discussion

This single‐center study of 80 consecutive adults with newly diagnosed CD describes the endoscopic and histopathological features of CD at diagnosis in a Vietnamese tertiary‐center population. The study was descriptive and had no internal or contemporaneous external comparator. Published data cited below are therefore given for context only, not as formal comparisons.

### 4.1 Time to Diagnosis and Disease Behavior at Diagnosis

In our cohort, the median time from symptom onset to diagnosis was 12 months (IQR 2–24). Before a definitive diagnosis, 78.8% of patients had undergone at least one colonoscopy (median 2 procedures). We did not assess the determinants of this interval; possible contributors include low disease awareness among primary care physicians, the overlapping clinical and endoscopic features of CD with intestinal TB in a high‐TB‐burden region, and the nonspecific endoscopic appearance observed in a proportion of cases. Diagnostic delay is a recognized global challenge, documented even in developed countries like Switzerland [11]. Without a comparator, we cannot tell whether this interval differs from that in other settings. A Chinese study linked diagnostic delay to a higher rate of subsequent surgery; our design did not allow this association to be examined [12].

At diagnosis, 34 patients (42.5%) had inflammatory (B1), 23 (28.8%) stricturing (B2), and 23 (28.8%) penetrating (B3) behavior. For orientation, inflammatory behavior accounted for approximately 74% of patients at diagnosis in a Western cohort [13]. Complicated behavior accumulates with disease duration, and the proportion with inflammatory disease fell to 12% after 20 years in another Western cohort [14]. Differences in referral pattern, case ascertainment, and study period preclude direct comparison with these cohorts. In addition, 20 patients (25.0%) had undergone intestinal resection before a definitive CD diagnosis, often for obstruction or suspected malignancy/TB. Similar observations have been reported from India, where IBD is also emerging. However, these descriptive data cannot show whether the time to diagnosis contributed to complicated behavior in our cohort [15].

### 4.2 Endoscopic Features at Diagnosis

Terminal ileal involvement (77.6%), cecal involvement (56.6%), and skip lesions (72.4%) were the most frequently recorded endoscopic characteristics, whereas hallmark morphological features were recorded in a minority of patients [8, 15, 16]. Longitudinal ulcers and cobblestoning, which are regarded as hallmark endoscopic findings, were recorded in 14.5% and 6.6% of patients, respectively [8]. Aphthous ulcers were recorded in 13.2%. Proportions of 60.1% and 54.7% have been reported in Korean and Indian cohorts, respectively, although with different designs and definitions [17, 18]. We do not consider that this pattern can be explained by disease stage alone. If advanced disease were the main reason, strictures and fistulas would be expected to be frequent endoscopic findings. In our cohort, luminal narrowing was seen in 32.9% and fistulous openings in only 5.3%. Yet 28.8% of patients had penetrating behavior, identified mainly by imaging or surgery. Several other, non‐mutually exclusive factors are likely relevant. First, one‐quarter of patients had undergone resection before diagnosis, so the most affected segments may already have been removed. Severe strictures also limited complete examination in some patients. Second, 11.3% of patients had received empirical anti‐TB therapy, which may have modified mucosal appearances. Third, the description of ulcer morphology (e.g., longitudinal vs. irregular ulcers) varies between observers. In the retrospective component, it also relied on stored images that may not have captured all lesions. Fourth, milder cases may have been managed as infectious colitis in primary care and not referred. Finally, true phenotypic differences cannot be excluded. Our descriptive design cannot distinguish between these explanations.

Anal canal lesions were identified in 15.8% of patients. This relatively modest proportion may partly reflect technical factors, as adequate visualization of this region requires careful and deliberate inspection. Landmark studies have demonstrated that longer withdrawal times significantly improve lesion detection during colonoscopy [19]. Applying similarly rigorous inspection techniques such as careful retroflexion and slow withdrawal through the distal rectum and anal canal is crucial to avoid missing perianal manifestations in CD.

Luminal narrowing was observed endoscopically in 32.9% of patients, and 28.8% had stricturing (B2) behavior. These measures are not equivalent. Montreal B2 requires constant luminal narrowing with prestenotic dilatation and/or obstructive symptoms, without penetrating disease. Endoscopic narrowing may instead reflect inflammatory edema, and patients with both narrowing and fistulas are classified as B3. We did not formally assess the relationship between these findings.

### 4.3 Diagnostic Uncertainty: Differentiating From Tuberculosis and Malignancy

The overall endoscopic impression was consistent with CD in 46.1% of patients; 35.5% showed nonspecific inflammatory changes, while 13.2% and 3.9% were initially suggestive of CRC and intestinal TB, respectively. These findings illustrate the diagnostic challenge in TB‐endemic regions where CD and intestinal TB share overlapping features including ileocecal involvement, ulceration, and stricture formation [20]. International guidelines emphasize systematic biopsy protocols and rigorous TB exclusion in such settings [8, 15]. Our experience reinforces that a high index of suspicion, combined with comprehensive microbiological and histological evaluation, is important to avoid both delayed CD diagnosis and inappropriate anti‐TB treatment. In addition to intestinal TB, differentiating CD from colorectal cancer (CRC) was also relevant in our cohort: 10 patients (13.2%) presented with lesions endoscopically mimicking CRC, characterized by ulcerated masses or significant luminal narrowing. Given the concurrent rise in CRC incidence in Vietnam, this phenotypic overlap poses a dual diagnostic dilemma. In these patients, multiple biopsies and careful histopathological assessment were used to distinguish inflammatory lesions from malignancy.

### 4.4 Upper Gastrointestinal Involvement

EGD was performed in 38 patients (47.5%) based on the presence of upper gastrointestinal symptoms, identifying lesions suggestive of CD in only 4 patients (5.0%). This low yield is consistent with current guideline recommendations. Although upper GI tract involvement can occur in up to 16% of patients with CD [21], routine EGD is currently not recommended in adult patients suspected of having CD; rather, it should be performed only when upper GI signs or symptoms are present or when colonoscopy is nondiagnostic [22]. Our findings support this selective, symptom‐directed approach: universal EGD in asymptomatic patients appears to offer limited additional diagnostic value in this population.

### 4.5 Histopathological Features and Sampling Limitations

Histologically, chronic inflammation was universal but often nonspecific. Basal plasmacytosis (73.4%) served as a useful marker of chronicity, while crypt architectural distortion (38.0%) and focal inflammation (26.6%) provided supportive evidence. Non‐caseating epithelioid granulomas, which are the most specific histological feature, were identified in only 31.6% of patients, consistent with the wide variation of approximately 13%–67% reported in the literature [23]. While this relatively low detection rate reinforces that granulomas cannot be relied upon for diagnosis, their presence warrants careful interpretation in a TB‐endemic setting. Crucially, specific morphological features aided in differentiation: the granulomas identified in our CD patients were predominantly small, discrete, and non‐caseating, distinct from the large, confluent, and caseating granulomas typical of intestinal TB. Although the detection of any granuloma triggered a rigorous screening protocol—including tissue PCR and, where necessary, a therapeutic trial—the absence of caseation combined with negative microbiological markers ultimately supported the diagnosis of CD. Thus, detailed morphological characterization served as a helpful discriminator rather than a confounding factor.

Although limited by the small number of surgical specimens (*n* = 7), our preliminary observation that transmural inflammation was significantly more frequent in surgical cases (85.7%) compared to endoscopic biopsies (30.6%, *p* = 0.007) aligns with expected depth limitations of endoscopic sampling, which typically captures only mucosa and superficial submucosa. As noted in the literature, transmural changes can only be identified in resection specimens, as biopsies are more superficial; biopsies are limited in determining the depth and distribution of inflammation, and diagnosis is often reliant on clinical correlation or examination of the resection specimen [24]. This highlights the complementary roles of endoscopic biopsy and surgical pathology in CD diagnosis.

Overall, histopathology was consistent with CD in 67.1% of cases but showed only non‐specific chronic inflammation in 30.4%. All included patients had confirmed CD. This proportion therefore reflects how often histologically distinctive features were present, not the diagnostic accuracy of biopsy. For patients demonstrating nonspecific inflammation, the diagnosis of CD was confirmed largely based on characteristic endoscopic features (e.g., deep longitudinal ulcers, cobblestoning, segmental distribution) and radiological evidence of transmural disease or skip lesions via CT/MR enterography, in accordance with ECCO guidelines. In these patients, the final diagnosis therefore relied on information beyond the index biopsy, in accordance with the ECCO guideline [8].

Structured diagnostic criteria offer an alternative to global expert assessment. Tanaka et al. proposed simple scoring criteria for colorectal biopsies. These use crypt atrophy or distortion, basal plasmacytosis with mononuclear cell infiltration, segmental crypt abnormalities and mucin depletion, and sarcoid‐like granulomas. In 117 patients with CD, 125 with ulcerative colitis, and 484 with non‐IBD colitis, sensitivity and specificity exceeded 90% for the “probable” categories [25]. However, the criteria were developed for colorectal biopsies from Japanese patients with active colitis, and they require several specimens including the rectum. Only three patients with intestinal TB were included. Their value in ileal disease, which predominated in our cohort, and in separating CD from intestinal TB is therefore not established. The Japanese diagnostic criteria define CD by major findings (longitudinal ulcer, cobblestone appearance, and non‐caseating epithelioid granuloma) and secondary findings. The secondary findings are extensive irregular or round ulcers or aphthae for at least 3 months, characteristic anorectal lesions, and characteristic gastroduodenal lesions. The criteria also list the conditions to be excluded [26]. Neither set of criteria was applied in this study, so their performance in our cohort is unknown. We recorded several of the histological features used by Tanaka et al. including crypt distortion, basal plasmacytosis, focal inflammation, and granulomas. Others, such as mucin depletion and Paneth cell metaplasia, were not recorded, so the score could not be calculated retrospectively. Standardized criteria might have reduced the proportion of non‐specific biopsies and improved reproducibility. However, longitudinal ulcers, cobblestoning, and granulomas were each found in a minority of our patients. We therefore cannot estimate how many patients would have met these criteria at the index evaluation. Prospective evaluation of such criteria in Vietnamese patients, including those in whom intestinal TB is suspected, is warranted.

### 4.6 Clinical Implications

These findings may have several practical implications. First, educational initiatives targeting primary care physicians and general gastroenterologists may help to improve CD recognition in emerging IBD regions [15]. Second, systematic biopsy protocols and cross‐sectional imaging (CT/magnetic resonance [MR] enterography) should be employed early in patients with chronic gastrointestinal symptoms to facilitate timely diagnosis [8]. Third, 57.5% of patients in this cohort had stricturing or penetrating behavior at diagnosis. Early consideration of effective medical therapy may therefore be warranted, although treatment was not evaluated in this study. Recent evidence suggests that the use of biologic agents earlier in the treatment course improves patient outcomes and can prevent progression to irreversible bowel damage; early combined immunosuppression has been shown to result in remission more quickly and at higher rates than conventional “step‐up” management [27].

### 4.7 Limitations

This study has several limitations. First, as a single tertiary center study, referral bias toward more severe cases is likely, thereby limiting the generalizability of the findings to the broader Vietnamese population. Second, the retrospective component limited standardization of some data elements. Third, dedicated pelvic magnetic resonance imaging (MRI) was not routinely performed in all patients; therefore, the true prevalence of perianal disease may have been underestimated. Fourth, the sample size of patients requiring upfront surgery was small (*n* = 7), meaning statistical comparisons of histopathological features between surgical and endoscopic specimens should be interpreted with caution as exploratory findings. Fifth, histological conclusions were based on expert global assessment rather than validated biopsy criteria. Likewise, endoscopic impression categories used predefined but non‐validated definitions. This may have affected the proportion of nonspecific cases and limits comparison with other studies. Sixth, the study had no internal or contemporaneous external comparator. References to published cohorts are therefore descriptive and do not show differences between populations.

## 5 Conclusion

In this tertiary‐center cohort of Vietnamese adults with newly diagnosed CD, the median time from symptom onset to diagnosis was 12 months. At diagnosis, 57.5% of patients had stricturing or penetrating behavior, and 25.0% had already undergone intestinal resection. Longitudinal ulcers, cobblestoning, and epithelioid granulomas were each found in a minority of patients, and histopathology was non‐specific in 30.4%. These descriptive data provide a baseline for future comparative studies and for prospective evaluation of standardized endoscopic and histological criteria in Vietnam.

## Funding

The authors have nothing to report.

## Disclosure

This manuscript was prepared in accordance with the STROBE guidelines for observational studies.

## Ethics Statement

The study was approved by the Institutional Review Board of the University of Medicine and Pharmacy at Ho Chi Minh City (approval number: 711/HDDD‐DHYD, date: 15 May 2023). Written informed consent was obtained from all prospectively enrolled patients; consent was waived for retrospective cases per ethics committee approval.

## Consent

The authors have nothing to report. The images are entirely unidentifiable and there are no details on individuals reported within the manuscript.

## Conflicts of Interest

The authors declare no conflicts of interest.

## Data Availability

The data that support the findings of this study are available from the corresponding author upon reasonable request.
